# Motivations for participation in nonprofit homeshare programs in the United States: a qualitative study with older home providers and home seekers

**DOI:** 10.1093/geroni/igaf141

**Published:** 2025-12-23

**Authors:** Susanna R Curry, Molly Calhoun, Angela K Perone, Leyi Zhou, Elizabeth Xanders Pinkis

**Affiliations:** School of Social Work, California State University, Sacramento, Sacramento, California, United States; School of Social Work, California State University, Chico, Chico, California, United States; School of Social Welfare, University of California, Berkeley, Berkeley, California, United States; School of Social Welfare, University of California, Berkeley, Berkeley, California, United States; Department of Public Health, California State University, Sacramento, Sacramento, California, United States

**Keywords:** Shared housing, Financial security, Homesharing, Housing insecurity, Social isolation

## Abstract

**Background and Objectives:**

Homesharing provides a strategy for addressing housing insecurity by pairing home providers, often older adults, with an extra room in their house with home seekers needing housing. Despite 5 decades of use, research on this intervention remains limited. This study aims to build on this sparse scholarship to provide insight into the motivations for participating as either home seekers or home providers.

**Research Design and Methods:**

This community-engaged qualitative project includes data from 24 in-depth interviews and short demographic surveys with a diverse group of home providers (*n *= 13) and home seekers (*n *= 11) recruited from 2 nonprofit homesharing organizations. Interviews were recorded and professionally transcribed. The researchers used constant comparison techniques to identify patterns and unique perspectives in the transcripts.

**Results:**

Home providers and home seekers had a mean age of 67.92 (*SD* = 9.39) and 52.09 (*SD* = 19.03), respectively and were racially/ethnically diverse. The overall sample was primarily female (71%), though more home providers were female (85%) than home seekers (55%). Participants described a range of motivations for participating in homesharing, including financial motivations, the desire for companionship, the result of a disaster or life change, the desire for a task exchange arrangement, the need for administrative/third-party support for housing (including a need for safety and security), and altruistic reasons.

**Discussion and Implications:**

This article provides important new data in a vastly understudied area that can inform policy and practice to support affordable housing options for older adults—particularly through nonprofit homesharing programs.

**Innovation and Translational Significance:** Homeshare organizations can support older adult homeowners who want to take in a roommate so that they can remain in their home. While the benefits of homesharing are well-documented, there is much less known about motivations for participation. This community-based qualitative study found that participants joined homesharing due to financial motivations, the desire for companionship, the result of a disaster or life change, the desire for a task exchange arrangement, a need for safety and security, and altruistic reasons. Findings from this study will help homeshare organizations further support potential and current participants as they aim to age in place.

Homelessness and housing insecurity are some of the most significant contemporary social problems and carry large political weight in many countries. Of particular concern is the growing proportion of people who are experiencing homelessness in older age. In the United States, older adults comprise one of the fastest-growing groups experiencing homelessness, representing nearly half of the people who are homeless (Henderson et al., 2023; Pearson et al., 2019). More broadly, older adults are the fastest-growing population in the United States, and their financial stability has significantly decreased over the last 20 years, particularly among older women and people of color (Joint Center for Housing Studies of Harvard University, 2024; Pearson et al., 2019).

The reasons for homelessness are vast; however, one of the greatest barriers to mitigating homelessness is the lack of housing (Colburn & Aldern, 2022). For older adults, there is also a lack of both affordable and appropriate housing that suits the needs associated with evolving abilities and needs over time (Pearson et al., 2019). Despite a lack of affordable housing, single-family homes are underutilized; just over 60% of homes across the United States have a spare bedroom (Ghilarducci, 2023).

## Homesharing

One promising strategy for addressing housing insecurity, growing care needs, and a desire to age in place or age in community among older adults is “homesharing” (Perone et al., 2025a). Non-profit homesharing organizations pair home providers, often older adults, with an extra room in their house as providers with “home seekers,” who need assistance finding housing they can afford. In some cases, home providers may charge below-market rate rent in exchange for care support from the home seeker, such as light housework, rides to appointments, or assistance with yard work (Magid et al., 2022; Martinez et al., 2020). While these “service exchange” models are common, some arrangements involve solely the exchange of rent for housing and do not involve an exchange of services, and some arrangements may involve a combination of rent and service exchange (Gutman et al., 1989; Perone et al., 2025a).

### Nonprofit homesharing organizations

Unlike traditional roommate arrangements, nonprofit homesharing involves the support of a formal nonprofit organization, which carefully vets individuals seeking to provide housing or live in someone else’s home, provides careful matching according to preferences and personalities, and supports matches in developing shared living and rental agreements to ensure ongoing sustainability of the arrangement (Perone et al., 2025a). The use of formal programs to assist with homesharing has existed in the United States since the 1970s (Gutman et al., 1989), and now exists internationally in over 16 countries (HomeShare U.K, n.d.).

The model can support older adult home providers who may be interested in taking in a roommate (with homeshare organization support) for additional income, companionship, or light assistance around the house to age in place. About 88% of older adults wish to stay in their home as long as possible (Institute for Healthcare Policy & Innovation, 2023), yet there are challenges to aging in place, including needing assistance with basic tasks to maintain the home. Some also see homeshare as a tool for homelessness prevention for “home seekers” of all ages (Perone et al., 2025a). Diverging from traditional crisis-centered approaches to homelessness, homeshare provides an option to home seekers who are precariously housed and on the verge of homelessness (Perone et al., 2025a).

Homesharing has been a housing tool for over five decades; however, research on this intervention has been limited. A recent scoping review cited sparse evidence on the model (Martinez et al., 2020). Generally, older adult home providers and home seekers report benefits including interpersonal and financial support, instrumental activities of daily living (IADLs), and a greater sense of security (Bodkin & Saxena, 2017; Magid et al., 2022; Martinez et al. 2020). There is some evidence that homesharing can reduce social isolation and loneliness by creating opportunities for interaction within the built environment (e.g., Lubik & Kosatsky, 2019). Homeshare participants have also reported reduced loneliness (Macmillan et al., 2018; Martinez et al., 2020) and isolation (Macmillan et al., 2018) and reported improved well-being (Rekart & Trevelyan, 1990).

While the benefits of homesharing are well-documented, there is much less known about what motivates home seekers and home providers to participate in the program initially, or what factors encourage them to continue. One study suggested that in intergenerational homeshare programs, home providers have expressed motivations related to altruism (helping young people find housing) or intergenerational learning (Labit & Dubost, 2016). However, a much broader exploration of home seeker and home provider motivations to participate is needed. This study is based on community-engaged qualitative analysis exploring the motivations for participation in homesharing across two programs in California. It contributes to a small but important body of scholarship on homesharing amidst growing concerns about housing affordability and aging in place for older adults.

## Methodology

This study utilized a community-engaged qualitative approach to explore the perspectives of home seekers and home provider motivations for participation across two different homeshare programs. Community-engaged research foregrounds shared power in decision-making (Perone et al., 2025b; Ubri et al., 2024), in which community partners are involved throughout the research process, including developing research questions (Owens et al., 2025), identifying culturally responsive recruitment approaches (Richardson et al., 2025), and producing research products that are readily accessible to the community partners (Heiden-Rootes et al., 2024). The research team regularly met with at least one community partner nonprofit homesharing organization to get input on research design, including recruitment strategies and community-driven research products. This article is part of a larger qualitative research study of homeshare programs in California, which received ethical approval by the University of California (IRB #2023-07-16550). The research question for this study was What motivations drive homesharing participants to engage in a nonprofit homesharing partnership?

### Sample and data collection

Participants were sampled through a purposive sampling strategy, wherein two homeshare organization directors partnered with the research team to provide the opportunity for participation to home seekers and home providers who then opted into the study. Those who opted in were contacted by a member of the research team to set up an interview. A total of 24 participants were interviewed for this study, with balanced representation between home seekers and home providers (see Table 1).

**Table 1.** Demographic characteristics of the study sample.

Characteristics	**Home seekers** ** (*n* = 11)**		**Home providers** ** (*n* = 13)**		**Total sample** ** (*n* = 24)**	
	*n*	*%*	*n*	*%*	*n*	*%*
**Age**						
** 50 and below**	5	45	0	0	5	21
** 51–60**	1	9	3	23	4	17
** 61–74**	4	36	5	38	9	38
** 75+**	1	9	4	31	5	21
** Missing**	0	0	1	8	1	4
**Race/Ethnicity**						
** White**	2	18	8	62	10	42
** Hispanic/Latino**	4	36	1	8	5	22
** Asian**	1	9	2	15	3	13
** Black/African American**	2	18	0	0	2	9
** Arab**	1	9	0	0	1	4
** Multiracial**	0	0	1	8	1	4
** Missing**	1	1	1	8	2	8
**Marital** s**tatus^a^**						
** Single**	5	45	7	53	12	50
** Widowed**	1	9	3	23	4	17
** Divorced**	2	18	0	0	2	9
** Partnered**	1	9	0	0	1	4
** Single and widowed**	0	0	1	8	1	4
**Divorced and widowed**	1	9	0	0	1	4
**Partnered, living apart together**	0	0	1	8	1	4
** Other (separated)**	1	9	0	0	1	4
**Gender^b^**						
** Woman**	6	55	11	85	17	71
** Man**	5	45	1	8	6	25
** Missing**	0	0	1	8	1	4
**Sexual orientation**						
** Straight/Heterosexual**	8	73	8	62	16	67
** Gay/Lesbian**	0	0	2	15	2	9
** Bisexual**	0	0	1	8	1	4
** Pansexual**	1	9	0	0	1	4
** Prefer not to say**	2	18	1	8	3	13
** Missing**	0	0	1	8	1	4
**Income**						
** $0–$19,999**	2	18	1	8	3	13
** $20,000–$39,999**	3	27	3	23	6	25
** $40,000–$59,999**	2	18	3	23	5	21
** $60,000–$79,999**	2	18	1	8	3	13
** $80,000–$99,999**	0	0	0	0	0	0
** $100,000–$119,999**	1	9	3	23	4	17
** $120,000 or more**	1	9	1	8	2	9
** Missing**	0	0	1	8	1	4

a Participants were asked to indicate “all that apply” regarding their marital status.

b No participants selected a gender identity other than “man” or “woman.”

Data were collected through in-depth semi-structured interviews (see Supplementary Material for the interview protocol) conducted via Zoom or in person. Prior to each interview, participants were asked to choose a pseudonym that would be used in the interview and in all dissemination of findings. Each participant was asked for verbal consent for participation and the recording of the audio transcripts. Participants were then asked a series of open-ended questions related to their motivations for participating in a homeshare arrangement (see Supplementary Material). Each interview lasted approximately 45–60 min. At the culmination of the interview, participants were asked to complete a self-report demographic survey through Qualtrics. Each participant received a $50 gift card in appreciation for sharing their experience and expertise.

### Data analysis

Each interview was audio-recorded and transcribed via Rev.com. Transcriptions were de-identified. Data analysis incorporated a hybrid approach to coding (Perone, 2023), including a five-step thematic analysis process of inductive and deductive coding as outlined by Bingham (2023), which began by organizing the data and ended with documenting the findings. Initially, each member of the research team was assigned four transcripts to code using a priori coding based on the interview protocol, and open coding strategies. A priori coding was the deductive strategy that set the frame for coding (Bingham, 2023). The inductive coding strategy explored the data for patterns and emerging themes that fell into the broader a priori codes (Bingham, 2023). The research team used constant comparison techniques, identified discrepancies, and came to an agreement (Bingham, 2023; Strauss & Corbin, 1998). Using this approach, the team created a codebook and coded each of the transcripts using the Dedoose coding software, version 9.2.22. After the first round of coding, two members of the research team conducted a second round of coding and noted patterns. Finally, the research team developed the major themes regarding the motivations that drive homeshare participants’ engagement in the program. To develop the themes, two members of the research team independently derived an initial description of each theme and utilized data to illustrate the similarities and differences in the patterns. The team then met to discuss and come to an agreement on the themes. To ensure rigor and trustworthiness, the research team used strategies such as thick description, memoing, and peer debriefing to minimize bias throughout the process (Bingham, 2023). Once the findings were outlined, the research team utilized current literature to explore where the research findings reinforced or added to previous research.

## Findings

Most of the participants in the homesharing programs were older adults, though the study also included some younger home seekers. The average age of participants was 60.4 (*SD* = 16.55), with an age range of 21–88. Home providers had a higher average age (mean 67.92, *SD* = 9.39) than home seekers (52.09, *SD* = 19.03). As shown in Table 1, the most common racial/ethnic identity for home seekers was Hispanic/Latino (36%), followed by White (18%) or Black/African American (18%). In contrast, home providers were primarily white (62%), followed by Asian (15%). Just over half of home providers (53%) and 45% of home seekers identified as single. Over half (55%) of home seekers and 85% of home providers identified as female. The majority of both home providers and home seekers (73% and 67%, respectively) identified their sexual orientation as “straight/heterosexual,” one home seeker identified as “pansexual” and two home providers identified as “gay/lesbian.” Incomes of home seekers and home providers varied, though home providers generally had higher incomes. Home provider incomes varied much more than home seekers; four home providers had incomes below $39,000, four had incomes between $40,000 and $79,000, and four had incomes above $100,000. Home seekers more generally had lower incomes, including five with incomes at or below $39,000, four between $40,000 and $79,000, and two above $100,000.

Home providers and home seekers described a range of motivations for participating in homesharing, including financial motivations, the desire for companionship, the result of a disaster or life change, the desire for a task exchange arrangement, the need for administrative/third-party support for housing (including a need for safety and security), and altruistic reasons. Below, we discuss these motivations and how they were similar or different among home providers and home seekers.

### Motivations for participation

#### Financial motivations

The financial impact of homeshare participation was a primary motivator across both providers and seekers. Many providers sought homeshare roommates to share the expenses of maintaining a home. The homeshare model sometimes allowed homeowners to maintain their house so that they did not have to sell it. For example, Ginger, a 66-year-old widowed woman, worried that selling her house would have priced her out because she would not be able to afford a smaller house due to changes in the market. She said about this:

I always said that I would only live in this house 10 years after my husband died, because it’s a huge home…it’s not so easy for people to downsize and find something that’s affordable. That kind of limits it. I don’t know where I’d be. Home sharing like this is probably going to, if I need to, give me the ability to stay in the home longer.

Here, Ginger suggests that she was not able to find an affordable way to downsize her housing, leaving her with a large house that she could not afford to keep without homesharing. Similarly, Mary shared that she was motivated to get involved in homesharing in order to stay in her home due to the costs associated with upkeep of her house:

I thought I didn’t make enough money, and I thought I got to do something fast, either sell the house or do something. But then [homesharing organization] really saved my neck.

Overall, home providers were motivated financially due to high mortgages, difficulty keeping up with the costs of maintaining a home, and life changes such as the death of a husband. Home seekers also had primarily financial motivations, though their financial needs were somewhat different. Many home seekers were previously in precarious housing arrangements and had been looking for an affordable place to live for some time. Many indicated that they had been looking for a place in the community where they worked but had found it difficult due to the cost of living. One of the factors that made it difficult for many home seekers to secure housing that was affordable was poor credit. Both Tanit and Carmen indicated concern about their credit scores and other financial requirements. For example, Tanit, a divorced woman in her 50s, explained her situation after securing a job in an expensive city but having difficulty finding housing that was affordable nearby:

One of the benefits of going through [the homeshare organization] is rather than do a credit check, they do a background check. And that was kind of one of my barriers, needing to have a very high credit score and three times the rent.

Similarly, Carmen, a 68-year-old woman, worried about the ability to secure an affordable place to live because of her credit. She explained:

I was worried about my credit because I didn’t think I would qualify with my credit in renting an apartment or something. That’s what worried me. But when [the homesharing organization] told me, “You don’t have to worry about your credit.”… I mean, for now, I needed it right away because I needed to get out from where I was. I wasn’t happy there. So it was like a God-saver for me.

#### Companionship

Both home providers and home seekers were motivated to join the homeshare program due to a desire for companionship, though home providers cited this as a primary or secondary motivation more often than home seekers did. A few home providers had recently lost a husband and shared that they wanted someone else in the home because they were not used to living alone. For example, Ginger found that after her husband died, she realized that she needed someone else in the home. She said about this:

When my husband died in 2017, it was the first time in my entire life I’d ever lived alone. I always had roommates. I sat with that for a while… That wasn’t my motivation. I thought my motivation was that… Well, and I do know that, I don’t want to live by myself.

While Ginger had multiple motivations, she shared that her need for companionship was an unexpected reason for joining and continuing in homeshare, even through multiple different matches. Similarly, Marie, a 56-year-old woman, found that after her mother died and she inherited her mother’s house, she realized that she did not want to live alone. She said, “I was just lonely too. I mean it’s just kind of creepy when you’re all by yourself, lonely and stuff.” For Marie, having someone else in the home provided both friendship and a feeling of security:

You feel more secure, you have a companion, and they watch out for you too, and I watch out for her. I think you’ve become very good friends, and ones that could stay on for a while. I think friendship can go on long.

While many of the home providers indicated wanting someone else in their home, fewer home seekers cited companionship or the desire for a relationship with their housemate. Out of the three seekers who cited the desire to have a connection with the person they live with, two were particularly interested in living with an older adult, with one being specific about helping the provider to stay in his home. For example, Ethan, a 63-year-old man, said:

Lord bless me, I had really intelligent parents, [could] carry on a conversation with a brain surgeon to a guy who drives a car. That doesn’t matter to me, talk to anybody. And so I like the idea of just A, helping somebody out. And then B, kind of a co-gen [intergenerational] relationship.

Ethan expressly desired an intergenerational relationship, making homeshare a good fit for him. Similarly, Sylvia, a 61-year-old home seeker, noted a desire to live with an older adult in particular. She said about this:

I think I appreciate living with an older person for many reasons. One is I’m a very quiet person and tidy, pretty tidy. I’m not a party person, so I was seeking a home that was quiet. And I think you typically find that more with older people.

#### Disaster or significant life change

Several providers and seekers were motivated to participate in homeshare due to acute life events such as the death of a spouse (most commonly among home providers) or a toxic living situation that required an immediate move (most common among home seekers). For example, home providers Charlotte and Sally had both lost their husbands about a year prior to their interview, and needed a roommate almost immediately. Home seekers Tanit and Phillip were both very precariously housed and had conflict in their prior housing situations, which led them to seek homesharing. Both had been aware of homesharing previously, but had not been ready to match with a home provider until their housing situation became untenable. Tanit explained that she had already been in touch with the homesharing organization previously, but:

Then the situation actually became toxic, and I had to leave immediately. The situation happened at around noon, and I was in my car at 3:00 and on the phone with [staff person at homeshare organization], and I was like, I don’t really have anywhere to go.

Similarly, Philip, an 81-year-old man, got back in touch with the homeshare provider as soon as he had to leave his prior housing situation:

I left the house where I had been living for two years and happily so because of a conflict with another member of the house. And I had to leave quickly. And because of that, I didn’t really have any other place to live.

In many of these situations, the life event created an almost immediate need for financial support to maintain their homes (providers) or for affordable housing (seekers). Additionally, a number of seekers and providers from rural Northern California were impacted by the Camp Fire that burned down the town of Paradise in 2018, including many homes.

#### Task exchange

Some home providers shared that, in addition to financial motivations, they also decided to participate in homesharing because they needed support with IADLs or minor help in maintaining their home. However, the need for support varied. Linda shared that she needed someone around the home to help her with minor daily activities:

I had to find somebody to move in because I can’t… I can’t put the dishes away… I don’t need personal medical help. I don’t need someone to touch me. But I might need someone to take a sweater off a high shelf or to bring in a package or to open a bottle like a pill bottle.

While Linda had specific daily needs that required help from someone living in her home, Ginger described her needs in a broader sense. She said, “Homesharing like this, is…giving [me] the ability to stay in the home longer, just because I can share some of the burden of the house.”

Linda and Ginger were clear about needing support around the house; however, Mary was less clear. While Mary, a 76-year-old woman, mentioned having had falls and having to move items to lower shelves, when we asked her if her need for help was a motivation for participating in homesharing, she replied:

If there’s a problem somewhere, I’ll ask. And if they want to, they can. But it’s not part of the duty of the renter.

Many home providers did not initially think about task exchange or care support when considering homesharing but later found the option of support to be useful. However, some home seekers discussed the fact that they were motivated to join a homeshare arrangement in part due to the option for task exchange. For example, Lisa, a 36-year-old woman, shared:

I originally saw this match, I want to say back in May, and I was like, “Wow, this is just right up my alley. I love the price range.” I like the fact that there was tasks involved. It also helps you to get to know the people that you’re living with because sometimes you can be such strangers to each other if there is not, I feel like there’s not a task involved. And it involved cooking and something that I love to do and walking a dog, I have some grand dogs and I miss them. And what a nice way to have the company of a pet without the extra super responsibility of actually owning a pet. And that’s what really got my interest in this particular [match].

For Lisa, the benefit of having reduced rent due to the task exchange option, along with the ability to get to know the home providers better due to the service exchange, was a win-win. Shiny One, a 60-year-old woman, was also drawn to the program due to the task exchange. She had previously been living with someone she was caring for, and around the same time when that person died, she was in contact with the director of the homesharing organization in her area. She shared about this:

Pretty soon I’ll be 62, and then it’ll be coming back to me [in Social Security payments]… Because until then, I don’t have a constant income. It’s very hard to find housing. But, I’ll help take care of someone until I die.

Shiny One was looking for a housing option that would allow her to continue in her role and have reduced-cost housing in exchange. For her, the “timing was right” to join the homeshare program.

#### Third-party support and safety needs

Both home providers and home seekers expressed that the administrative support of the homeshare organization was helpful. The role of the administrative support of the third party was critical to many home providers who cited the advertising, vetting, background-check process, and the ability to have month-to-month arrangements associated with the homeshare organizations. Several providers had prior experience seeking roommates on Craigslist but had fears or concerns about using that service and preferred the homeshare organization, which felt more trustworthy. For many, the third-party support provided a greater sense of safety and security than they would have had otherwise. For example, home provider Rob, a 25-year-old man, said:

Background checks were a great tool. I know they’re expensive, so the fact that they were free, even if you didn’t end up using their services. And then, how they would check income, as well, just to make sure that somebody does have a steady kind of job. And just that mediation thing the…is just amazing because as somebody that’s freshly, relatively new renting to a person that doesn’t know, it’s hard to establish boundaries and make sure what’s correct to say to also make sure that you’re not just taking advantage of the other person as well as being taken advantage of.

Rob felt that the administrative support made him feel more confident about living with a stranger who had been vetted by a reputable organization. For another home provider, Italian Greyhound, an 82-year-old woman, the administrative support was helpful because:

They do everything for you. I was so used to doing everything around finding someone, the internet search and the marketing and references and all of that, and they do everything. So it was just such a relief.

Several home seekers also cited that the background check and pre-screening conducted by the homeshare organization made them feel more secure. Tanit found that the third-party support made her feel more secure in moving in with someone new:

I used the roommate, I think it’s called Roommates.com or something, and that was a little scary, but I think the thing about the [homeshare organization] is it provides an intermediary, two strangers coming together, but at least the homeowner knows that the other person’s been checked out, and the renter knows that the homeowners been checked out and then they can act as kind of an intermediary in case there’s ever any conflict. So I felt like that made me feel safe, especially as a woman. And so yeah, that scaffolding was definitely more appreciated than just the random housemate.

Veronica, a 22-year-old home seeker who had just graduated from college, also noted that she had tried to find a place to live online and that she felt unsure about whether to trust people she found on those sites. She explained that when she graduated, she:

Immediately went to online websites like apartments.com, Roommate Finders, Facebook Marketplace, all those kinds of things to find any room listings. And I did speak to a few people, tried to ask more questions about some things, but honestly, with online listings, it can be very suspicious and you never know if a person is true or not.

#### Altruistic reasons

A few homeshare providers expressed that they were driven to participate in homesharing by a need to help others in their community. For some, this meant being part of the housing crisis solution by providing affordable housing to those who need it. They cited their belief in the community and accessibility for their community, helping professionals who otherwise wouldn’t have access to live in the community where they worked. For example, Charlotte, a 56-year-old woman, shared,

I feel very strongly about low-cost housing in [town] for which we have hardly any. And so I was trying to figure out how I could find somebody that was local that couldn’t afford to live in [town], but worked in [same town]. I was interested in teachers, firefighters.

For Charlotte, one motivation was to help with the dearth of affordable housing available to people who contributed to her community. For Ginger and Ann, this meant providing housing to people who would otherwise be unhoused. Ginger said about this, “I have this huge house and the unhoused population is out there, and it just seems wrong. It seems wrong for me to have this big old house and not share it with somebody.” Similarly, Ann, a 78-year-old woman, said:

I mean, it’s nice to have extra income, but it’s mainly for… to assist in the homeless issue. I mean, not that I hired people off the street or anything, I mean, let tenants in off the street, but everybody’s potentially homeless if you can’t afford rent. And so this is just another option for people to… who can’t afford to have an apartment to live in community. But probably a stronger reason is I do believe in community.

For Ann, there were multiple motivations: the extra income that she needed for herself, the ability to help individuals in need of housing, and her community.

Shiny One identifies as a caregiver, which led her to participate in homeshare as a home seeker. She had been back in contact with the director of the homeshare program around the same time that she was looking for a new place to live after an older woman she had been caring for had died. When we asked her why she decided to be a part of the homeshare program, she said, “Well I’m a caregiver.” Later, she elaborated about her desire to be a part of something that helps older people stay out of institutionalized care, because:

When there’s people that are taking care of 16 people, they do not get the care. This program is a gift to seniors. It really is, because they don’t deserve to be thrown into places where nobody’s happy because of how hard they have to work, and it’s not going to get better.

## Discussion

This paper presents findings from a qualitative study exploring motivations to be involved in a non-profit homesharing organization as a “home provider” or “home seeker.” We found that the primary motivation for both home providers and home seekers was financial, but that there were many other motivations for both groups.

While most older adults own their home outright, about 37% of older adult homeowners have a mortgage, and these rates are higher among Black and Hispanic older homeowners (Butrica & Mudrazija, 2016; Herbert & Molinsky, 2020; Joint Center on Housing Studies, 2024). Older adults on fixed incomes, including those with and without a mortgage, may find it challenging to keep up with home maintenance and rising costs of utilities and property taxes (Fenelon & Mawhorter, 2020). Some providers in the current study were “property rich, cash poor,” meaning they owned a home with multiple bedrooms but did not have a large income to meet their monthly needs or maintain the home. Thus, it was not surprising that, for most of the home providers in the study, financial needs were a primary motivation to be involved in a homesharing organization. While none of the participants explicitly used the phrases “age in place” or “age in community,” many providers described a desire to seek supports that help them remain in their home as long as possible, including financial support or task exchanges.

Given the rise in the costs of housing for renters, most home seekers also expressed that their financial needs were a primary motivator for involvement in homesharing. Many had experienced significant and ongoing housing precarity prior to involvement in homesharing.

These findings expand on prior research exploring the role of financial needs in homesharing. While this study found that financial motivations were primary for both home providers and home seekers, prior literature regarding the role of finances for home providers is mixed. Bodkin and Saxena (2017) found that home providers were motivated due to the relative affordability of receiving assistance with household tasks by a home seeker in a homesharing arrangement, compared to paying for a ­caretaker. A scoping review of homesharing research (Martinez et al., 2020) cited three studies noting that the older the home provider was, the less likely they were to self-report a financial motivation or benefits of participation (Altus & Mathews, 2000; Macmillan et al., 2018; Rekart & Trevelyan, 1990). It is possible that motivations among home providers have changed over time as the context of housing and care for older adults in the United States has evolved. It is estimated that by 2029, 54% of older adults will be unable to pay for care associated with assisted living facilities or other housing for older adults (Pearson et al., 2019), particularly those who are middle-income.

Older adults are more susceptible to fraud attempts than individuals who are younger (Holtfreter et al., 2014). Many of the home providers in the study were motivated to be involved in homesharing, in part, because of the need to have a tenant, but had fears around how to do so without being scammed. These findings mirror two other study findings that indicated that home providers felt a greater sense of safety and security through homesharing (Labit & Dubost, 2016; Rekart & Trevelyan, 1990).

Many home seekers also indicated that they benefited from the security associated with support from the non-profit homesharing organization. In particular, many home seekers themselves had experienced fraud or difficulty finding a trustworthy roommate, and the background checks on both parties and other screening and matching support provided a sense of safety. While in this study, participants’ motivations related to help from a third party centered on assistance with matching, other prior research suggest that some participants desire and benefit from assistance with mediation related to relational conflict or other challenges, though not all need that level of support (Bodkin & Saxena, 2017; Labit & Dubost, 2016; Martinez et al., 2020). The findings from the present study emphasize the importance of building trust between the homeshare organization staff and potential or current participants early on.

Nearly 30% of adults 50–80 years old report social isolation, and 33.4% report that they feel a lack of companionship “some of the time” or “often” (Malani et al., 2025). Loneliness is most commonly reported among those living alone and those with lower household incomes (Malani et al., 2025). Companionship was an important motivator of homesharing participation among home providers in the current study, and some home providers indicated that they spent considerable time with the home seeker. Home seekers also benefited from the company of the home provider, sharing that they had developed friendships. These findings expand upon prior literature on the impacts of homesharing, providing further evidence that homesharing may reduce feelings of loneliness (Macmillan et al., 2018; Martinez et al., 2020; Rekart & Trevelyan, 1990).

Some home providers also indicated that they were motivated to be involved for altruistic reasons. When older adults leave the workforce, they may lose resources provided by their jobs or prior roles, including social status (Wetzel & Mahne, 2016). Taking on a role that is meaningful, such as volunteering, mentoring, or “hosting” in a homeshare organization, may improve feelings of meaningfulness and contribute to general well-being (van Ingen & Wilson, 2017). While it was less common, some home seekers also expressed a desire to be involved in task exchange and have an intergenerational relationship. Altruism is a less commonly discussed motivation or benefit in prior literature, with only one other study focused on intergenerational homesharing, finding that older adults expressed a desire to assist students who needed housing that is affordable (Labit & Dubost, 2016). The findings in this study build our understanding of the role of altruism in homesharing beyond purely intergenerational contexts, suggesting that some home providers are motivated to help others through homesharing.

It is important to note that this study is based on a small sample of home providers and home seekers in two homeshare organizations in California. California is at the forefront of the affordable housing crisis in the United States, and that reality may have influenced the financial motivations for providers and seekers to be involved. It may be that homeshare participants in relatively less expensive areas may emphasize other needs more strongly, such as needs for task exchange or companionship broadly defined. The sample was also primarily female, and there may be differences in motivation to engage in a homesharing arrangement by gender. This is also a point-in-time study, whereas participants’ perceptions of their own motivations for engaging in homeshare may be subject to recall bias depending on the length of the homeshare relationship.

This study was initiated through collaborations between university researchers and homeshare organizations seeking to build knowledge about the experience and value of homesharing. Findings from this study will help homeshare organizations further develop their recruitment and support to potential and current participants. In particular, the study builds practical knowledge around the importance of steps some homesharing programs take to reduce barriers to housing (including the lack of a required credit check for home seekers), as well as strong support by homeshare organizations in building trust. It also provides nuance to narratives that have historically indicated singular motivations for participation, clarifying that many participants have multiple strong motivations in addition to financial incentives to participate. However, further research is needed in several areas. In particular, further knowledge is needed on the role of policy in shaping home providers’ motivations to be involved in homeshare and the role of task exchange in these arrangements. Additionally, the costs and benefits of homesharing related to housing affordability models and aging in place are yet to be known. The relationship dynamics between housemates, particularly as they relate to task exchange in general, are important to explore. Finally, there is a significant need to understand the impact of homesharing on the social isolation of older adults.

## Supplementary Material

igaf141_Supplementary_Data

## Data Availability

Interview and survey data are not publicly available. Individuals interested in secondary data analysis of the data may contact the corresponding author.
