# Supplementary material for: Motivations for participation in nonprofit homeshare programs in the United States: a qualitative study with older home providers and home seekers
Source: Innov Aging. 2025 Dec 23;10(2):igaf141. doi: 10.1093/geroni/igaf141 (PMC12863404; doi:10.1093/geroni/igaf141)
Supplement: igaf141_Supplementary_Data [file igaf141_supplementary_data.zip › innage suppl Curry, Calhoun, Perone, Zhou, & Pinkis.docx]

***Innovation in Aging* Supplementary Material: Curry, Calhoun, Perone, Zhou, & Pinkis. Motivations for Participation in Nonprofit Homeshare Programs in the United States: A Qualitative Study with Older Home Providers and Home Seekers.**

**Home Share Research Interview Questions**

**Welcome/Introduction:**

I’d like to learn more about you as a person. Can you share a bit about how long you’ve lived in the area and what your interests are?

**Motivations:**

- How did you learn about [homesharing organization name]?
- Why did you decide to seek a home (or provide a home) for your home share program?
- What motivated you to make the decision when you did?
  - In what ways was your family/friends involved in this decision, if at all?
- What concerns did you have as you entered the home share program?
  - *Listen and probe for*: issues of agency, privacy
- How did you address these concerns?

**General:**

- How long have you been participating in [homesharing organization]?
  - Has it been continuous? Were there interruptions (if so, why and when?)
- What type of housing are you in?
- Who else lives in your home?
- Tell me about your experience with the program.
- Tell me about your living situation
  - Listen and probe for: Shared space, private space
  - How does this differ from prior living situations?

**Relationship Dynamics & Caregiving:**

- Tell me about your relationship with the home provider/seeker.
  - What benefits do you get from this relationship?
  - What challenges have you found with this relationship?
- Can you tell me about the shared expectations you have in this arrangement? (clarifying prompt if needed: for example, do you share household chores?)
  - How did you come up with these shared expectations?
- Do you spend time together?
  - What do you do together?
  - How many hours/week do you spend time together?
  - How do you feel after you have spent time together?
- How did you agree on the payment received/paid in exchange for housing?
  - How do you feel about this amount?

**Role of the 3rd Party:**

- Tell me about your relationship with about [homesharing organization]
- Who do you have the most contact with? What is the nature of that relationship?
- How did you find out about this program?
- What types of support do you receive from them?
- What do you wish was also provided through this program? Or what are the gaps in the program?

**Impact of Covid-19 pandemic**

- How has the pandemic shaped your experience seeking/providing housing in this model?

**Wrap-Up/Closing Questions**

- Why do you think home sharing is a good model for this area?
- Why do you think home sharing might not work in this area?
- If you weren’t in a home sharing program, where would you be?
